# Long-term electromyography-based virtual reality training for chronic upper-limb motor paralysis after stroke: an exploratory single-case experimental design

**DOI:** 10.3389/fresc.2026.1946303

**Published:** 2026-09-23

**Authors:** Yohei Nagaoka, Shinya Suzuki, Tsuyoshi Nakajima, Hiroyuki Ohtsuka, Akimasa Hayashi, Max Ortiz-Catalan, Yukari Ohki

**Affiliations:** 1 Department of Integrative Physiology, Kyorin University School of Medicine, Tokyo, Japan; 2 Department of Sport and Medical Science, Faculty of Physical Education, Kokushikan University, Tokyo, Japan; 3 Department of Rehabilitation, School of Nursing and Rehabilitation Sciences, Showa Medical University, Kanagawa, Japan; 4 Prometei Pain Rehabilitation Center, Vinnytsia, Ukraine; 5 Remedi Ukraine, Vinnytsia, Ukraine

**Keywords:** clinical feasibility, effectiveness, myoelectric pattern recognition, rehabilitation, safety, visual feedback

## Abstract

**Introduction:**

Stroke commonly causes various symptoms, such as motor paralysis. Limited motor recovery during the chronic stage of stroke is a challenge in rehabilitation. Motor training using myoelectric pattern recognition (MPR) and virtual reality (VR) may improve maladaptive sensorimotor integration, thereby promoting motor recovery after stroke (MVR training). We evaluated the feasibility of adding upper-limb MVR training to conventional rehabilitation therapy for >3 months in individuals with chronic motor paralysis after stroke.

**Methods:**

Two individuals with chronic stroke participated in this preliminary, single-case study. An open-label, repeated-measures design was used for Participant A, and an ABA-type single-case experimental design was used for Participant B. During the intervention period (Participant A: 40 sessions over 8 months; Participant B: 20 sessions over 3 months), participants underwent MVR training in addition to conventional rehabilitation care. During MVR training, the participants performed the posture-matching test (30 min, twice a week), requiring them to voluntarily move their paretic hand with real-time visual feedback of the virtual hand that was controlled by the MPR of the paretic upper-limb muscles.

**Results:**

Both participants completed the planned training sessions without serious adverse events. The Fugl–Meyer assessment score of the upper extremities was used to assess upper-limb motor function. Following VR training, the improvement from baseline exceeded the estimated minimal clinically important difference in Participant A (+7 points) but not in Participant B (+5 points).

**Discussion:**

In conclusion, these findings suggest that long-term MVR training is feasible and can be implemented without serious adverse events in individuals with chronic motor paralysis after stroke. Although improvement in upper-limb motor function was observed in one participant, further studies with larger samples and controlled designs are warranted to assess the efficacy of MVR training.

## 1 Introduction

Stroke is one of the primary causes of death and disability globally according to recent findings from the Global Burden of Disease Study (1). Motor paralysis is a frequently occurring persistent sequela after stroke (2–4), which impairs daily life activities and negatively impacts quality of life (5). Although motor function typically improves to some extent after stroke, spontaneous recovery is inherently limited. Particularly, among patients in the chronic phase (approximately 3–6 months after stroke onset), the effects of conventional rehabilitation are often insufficient (6–8). Intensive exercise training can temporarily improve motor function in the paretic limbs, even during the chronic phase (9, 10). However, sustained intensive, long-term rehabilitation is challenging in the chronic phase because of reduced motivation, dependence on healthcare professionals, and stroke-related functional impairments (11, 12). Although intensive, high-dose rehabilitation may benefit these patients, the social resources available to provide such interventions are generally limited to long-term healthcare systems.

Virtual reality (VR)-based visual feedback techniques are being increasingly developed for stroke rehabilitation (13–17). For example, commercially available gamified VR systems can accommodate a broad range of users while enhancing motivation (18–20). Because these systems are relatively easy to implement clinically, they are used as an adjunct to conventional rehabilitation (19, 21, 22). Compared with conventional rehabilitation alone, these systems increase the frequency of paretic limb use, training intensity, and training frequency during therapy (23–25). However, conventional VR systems, which typically rely on motion capture of body kinematics for input, are generally unsuitable for individuals with moderate-to-severe paresis.

VR systems differ basis the control modality used to interact with the virtual environment. In 2014, Ortiz-Catalan introduced myoelectric pattern recognition (MPR) to enable phantom motor execution as a rehabilitation approach for pain management in individuals with limb amputation (26). This approach captures electromyographic (EMG) signals from multiple muscle groups while patients attempt predefined movements. A machine learning algorithm is then trained to distinguish the myoelectric patterns associated with different movements (27). Once trained, the system recognizes attempted movements, which are then used to control virtual elements within the VR environment (28). Integrating these technologies enables serious gaming, which can improve therapy adherence and increase treatment dosage. Simpler EMG-based approaches have also been explored for stroke rehabilitation, in which a single EMG signal is used to control a single action within a virtual environment (29). Unlike conventional EMG systems, MPR uses multiple EMG signals to control multiple actions, thereby increasing the number and complexity of decoded movements. Moreover, short-term intensive training combining MPR, VR, and serious gaming has demonstrated clinically meaningful improvements in upper-limb motor function in individuals with chronic stroke (30). These findings suggest that MPR-based VR systems could expand access to VR rehabilitation through myoelectric VR (MVR) training. Although the intervention described above produced beneficial outcomes, its high intensity may limit its feasibility for some individuals with chronic stroke (31). In addition, the characteristics of patients most likely to benefit from MVR training have not been adequately established. Furthermore, previous MVR training interventions have been limited to relatively short durations. Consequently, the feasibility, safety, and effectiveness of long-term MVR training within rehabilitation care systems for individuals with chronic stroke remain unclear. Addressing these knowledge gaps may help identify sustainable rehabilitation strategies that maintain long-term therapy engagement. Therefore, this study investigated the preliminary feasibility and safety of long-term (>3 months) MVR training for upper-limb motor impairment in individuals with chronic stroke receiving rehabilitation in long-term care settings. Due to the exploratory nature of this study, clinical effectiveness was not determined; however, its effects on upper-limb functions were preliminarily assessed.

## 2 Materials and methods

### 2.1 Ethics statement

This study was conducted in accordance with the principles of the Declaration of Helsinki and all relevant guidelines and regulations in Japan. The study protocol was approved by the Faculty of Medicine Research Ethics Committee of Kyorin University (approval number: H30-092) and registered in UMIN-CTR (ID: UMIN000050915). All participants provided written informed consent before enrollment.

### 2.2 Population

Participants were prospectively recruited from among those who received daycare rehabilitation services at Honmoku Hospital, Japan. Candidates were screened using electronic care records. The inclusion criteria were as follows: (1) first symptomatic stroke of ischemic or hemorrhagic etiology, (2) > 6 months after stroke onset, and (3) age >40 years. The exclusion criteria were as follows: (1) musculoskeletal disorders of the affected upper limb, (2) visual deficits, and (3) severe cognitive impairment with a Montreal Cognitive Assessment score <25. We contacted candidates who met the criteria and enrolled them after they provided written informed consent. In the preliminary session at enrollment, we further screened candidates using a test of motor state decoding from EMG activity to evaluate their capacity to perform MVR training. No baseline measure was considered for eligibility assessment.

### 2.3 Design

This was an exploratory, hypothesis-generating study and employed a prospective, open-label, exploratory, preliminary, single-case experimental design. Since the study was designed as a feasibility and proof-of-concept investigation rather than an efficacy trial, two participants (A and B) underwent MVR training. For Participant A, who was the first participant in this study, a before–after comparative design was used because the feasibility and safety of MVR training in individuals after stroke were not yet established. The participant therefore served as an initial exploratory case to assess feasibility and inform the subsequent study design, rather than as a formal single-case experimental comparison. The intervention comprised a single phase of MVR training with conventional rehabilitation care (MVR + rehab) (40 sessions over 8 months). Conventional rehabilitation was provided throughout the intervention because the participant was recruited from a population receiving daycare rehabilitation services. Effectiveness was assessed at enrollment (month 0) and every month thereafter (0–8 months).

Participant B was recruited after Participant A completed the intervention. Since it remained unclear whether the observed effects in Participant A were due to concomitant MVR training with conventional rehabilitation care, an ABA design was used for Participant B. The intervention period comprised three phases: rehab alone (8 sessions for 1 month), MVR + rehab (20 sessions for 3 months), and rehab-carryover (8 sessions for 1 month). Similar to Participant A, conventional rehabilitation care was provided at all phases. For MVR + rehab, the participants underwent MVR training concomitantly with conventional rehabilitation care. For rehab alone and rehab-carryover, the participant received only conventional rehabilitation care. The order of the intervention phases and commencement time of each phase were not randomized. Effectiveness was assessed at enrollment (month 0) and every month thereafter (0–5 months). Owing to clinical constraints, the number of baseline data points was limited, and baseline stability could not be fully established. The participants, investigators, and assessors were not blinded to the intervention, phase, or session number. Therefore, this study should be interpreted as a preliminary hypothesis-generating investigation, and causal inferences regarding the effects of the intervention are limited.

### 2.4 Interventions

The training sessions were performed approximately twice per week at the daycare rehabilitation facility of Honmoku Hospital, Japan, under the supervision and guidance of an experienced physical therapist. The duration of each session was approximately 30 min each for MVR training and conventional rehabilitation care. For each session of MVR + rehab, MVR training was performed after conventional rehabilitation care on the same day.

#### 2.4.1 MVR training

##### 2.4.1.1 Equipment and setup

We used an MPR- and VR-based system consisting of a portable multichannel EMG recording device (8 channels, bandpass filter at 20–400 Hz, gain of 2,000, sampling rate at 2,000 Hz, 14 bits analog-digital converter) (32), a PC running BioPatRec (https://github.com/biopatrec/biopatrec), and MATLAB (The MathWorks Inc., MA, US) for visual feedback, signal processing, and task implementation (33). The present system uses a conventional laptop display without a head-mounted display and can therefore be considered a non-immersive VR configuration. Figure 1 shows a schematic illustration of the training setup. The participants were seated comfortably on a chair with a backrest. The paretic arm was positioned on an armrest without orthotic restrictions. The monitor on the laptop computer was positioned on a table in front of the participant. Surface EMG was performed on eight muscles of the affected upper limb: the biceps brachii, triceps brachii, brachioradialis, flexor carpi radialis, flexor digitorum superficialis, extensor carpi radialis, extensor carpi ulnaris, and extensor digitorum communis. A pair of adhesive Ag/AgCl electrodes (F-150S; Nihon Kohden, Tokyo, Japan) was placed on the skin over the belly of the respective muscles at an inter-electrode distance of 2 cm after skin preparation with alcohol.

**Figure 1:**
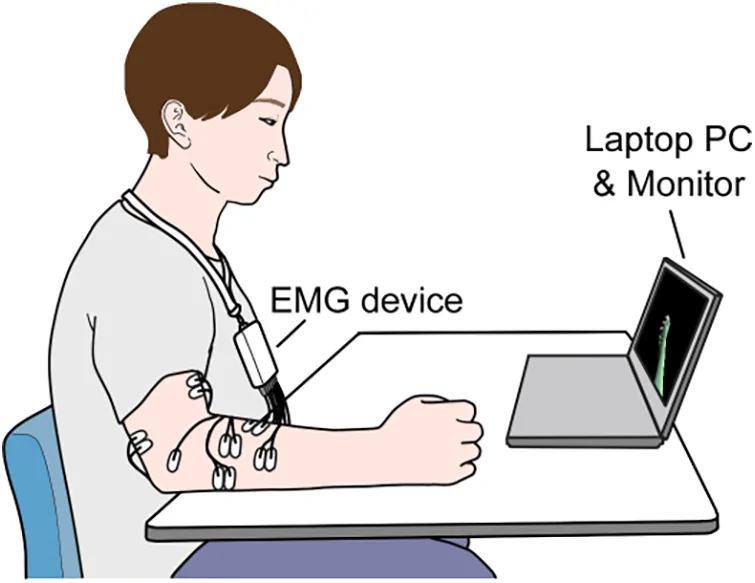
Schematic illustration of the training setup. The participants were seated comfortably on a chair. A conventional laptop personal computer and display were positioned on a table in front of the participant. Surface electromyography (EMG) was performed on eight muscles of the affected upper limb using a portable multichannel EMG recording device.

##### 2.4.1.2 Procedure

The general and detailed procedures have been described previously (26, 28). Each MVR training session consisted of three steps: recording, offline MPR training, and a posture-matching test. We targeted the wrist dorsiflexion/palmar flexion and hand opening/closing movements of the affected side.

First, we recorded the EMG activities of the eight test muscles during the target wrist and hand movements (test movements) described above to calibrate the VR system and construct a motor-intent classifier. After EMG electrode placement, the participants were asked to perform four types of test movements on the affected side. In the recording session, participants were asked to perform either two types of movements (single task: wrist dorsiflexion/palmar flexion or hand opening/closing) or all four (mixed task: wrist dorsiflexion/palmar flexion and hand opening/closing), according to the instructions of the model on the monitor. For each test movement, the participants performed three repetitions consisting of a 3-s contraction period followed by a 3-s relaxation period.

Second, we constructed classifiers of state-specific EMG patterns using a multilayer perceptron (MLP) classifier within the MPR framework described previously (27). From the pre-recorded, segmented EMG data, four signal features (mean amplitude, standard deviation, waveform length, and cardinality) were extracted for each muscle and each test movement every 200-ms time window (overlapped for 50 ms). The extracted datasets were then randomly assigned to training (40%), validation (20%), or testing (40%). Using the datasets for training and validation, the classifier was trained iteratively and refined using the MPR algorithm to decode the motor states and rest (e.g., rest, wrist dorsiflexion, and wrist palmar flexion). Finally, classification accuracy was calculated using the testing dataset to confirm adequate decoding of the selected motor states before proceeding with the motor-training exercises. When the decoding accuracy was considered insufficient (accuracy scores <75 for all types of test movements), the decoding procedure (i.e., EMG-recording and classifier-construction sessions) was repeated once again before training.

Third, participants performed a posture-matching test that required them to control virtual hand movements, which was achieved by real-time motor-state decoding using pre-refined EMG pattern classifiers (Supplementary Video S1) (27, 28, 34). When the EMG pattern was classified as a given state, the virtual hand moved in the relevant direction by 2° for each classification. The classification was updated every 50 ms (at a 20 Hz time-resolution). Typically, a single test run included 12 repetitions of two antagonistic movements at a single joint (wrist dorsiflexion/palmar flexion or hand opening/closing; two test movements×six repetitions), requiring control of a virtual hand movement with 1° of freedom (a single wrist/hand task). Each trial was initiated by presenting two virtual hands (the model and avatar) superimposed on the monitor. After short time intervals (∼3 s), the hand of the model quickly moved to the target position at 40° from the initial position. The participants were asked to move the avatar's hand volitionally immediately after the model's hand position changed and to match the avatar's hand position to the target. The task was considered complete when the hand of the avatar reached the target and maintained its position within the target for 2-s. The repetition was stopped if the participant did not complete the task within 30 s (33). During the posture-matching test, the therapist provided verbal encouragement and feedback regarding muscle activation, such as avoiding excessive muscle contraction and gradually increasing muscle activation. The therapist also monitored the participant's condition and interrupted the training to provide rest when necessary.

The number of test runs progressively increased across sessions. No predefined criteria for progression of task difficulty were established, and the task difficulty was not systematically increased during the intervention period. In the later sessions, the participants additionally performed a mixed wrist and hand task that required them to control a virtual hand movement with 2° of freedom and match the virtual hand position to four alternative target positions (wrist dorsiflexion/palmar flexion or hand opening/closing). In the mixed task, participants were required to complete 24 repetitions of the test movements in one test run (four test movements×six repetitions). The order of the target positions was randomly selected. Consequently, the number of trials for each session depended on the number of repetitions in a single test run, the trial completion time, and the completion rate.

#### 2.4.2 Conventional rehabilitation care

For conventional rehabilitation care, therapeutic exercises were administered for approximately 30 min. The exercises included relaxation, stretching, balance, standing, and walking, which were based on clinical practice guidelines for stroke rehabilitation (35–37). Relaxation and stretching exercises were also applied to the upper limbs. However, no active motor exercises for the upper limbs were provided.

### 2.5 Assessments

#### 2.5.1 Implementation status and safety

To monitor the fidelity of the training protocol, the investigator was asked to record whether the session was implemented as planned. In addition, during the MVR + rehab phase, the number of test movement repetitions also increased. We shared this information every month and pooled it at the end of the intervention period.

We expected that the intervention in this study would be safe and tolerable because of its noninvasiveness and the few mild adverse events (AEs) reported by previous studies on stroke rehabilitation using existing VR systems (38). Safety was assessed by continuously monitoring the occurrence of AEs and device failure during the intervention period. The seriousness and severity of AEs were defined in accordance with the guidelines of the International Council for Harmonization of Technical Requirements for Pharmaceuticals for Human Use (39). According to this guideline, an AE was considered to be any unfavorable and unintended sign (such as an abnormal laboratory finding), symptom, or disease temporally associated with the use of the device, whether or not it was considered related to the device.

#### 2.5.2 Offline decoding accuracy

We quantified the accuracy of the offline EMG pattern recognition using an accuracy score, defined as the percentage of correct classifications among the total number of classifications required to obtain 20 correct classifications. Although the accuracy score may vary depending on the electrode placement and impedance, higher values indicate greater classifier performance in predicting motor states and improved controllability of avatar movement. The accuracy score was computed using the test datasets for each test movement and task. The reference accuracy score was set to 75%, as required to control the system during subsequent training steps (30). Pearson's correlation analysis was performed to investigate temporal changes in the accuracy score across sessions in the MVR + rehab phase. The statistical significance level of the correlation coefficient was set at 0.05.

#### 2.5.3 Perceptions of avatar control

To evaluate the participants’ perceptions of avatar control, we obtained open-ended, verbal feedback from participants in sessions involving MVR training.

#### 2.5.4 Clinical effectiveness

An experienced physical therapist assessed the clinical effectiveness of the interventions using the following measures.

##### 2.5.4.1 Fugl–meyer assessment of the upper extremity (FMA-UE)

Motor function of the affected upper extremity was assessed using the FMA-UE motor score (40). Motor items included the upper extremities (0–36 points), wrist (0–10 points), hand (0–14 points), and coordination/speed (0–6 points). The examiner instructed the participants to perform each test movement and monitored whether they performed the movements correctly. The test movements were recorded using video cameras for offline scoring. The examiner scored the recorded movements. The total motor score was obtained by the sum of the four motor items (0–66 points). The participants were also evaluated for somatosensory function using the FMA sensation score, which consists of light touch (0–4 points) and position (0–8 points). The total sensation score was obtained by the sum of the scores of these items (0–12 points). Lower test scores indicated severe functional impairment. The minimal clinically important difference (MCID) of the FMA-UE motor score is estimated to be 5.25 points (41).

##### 2.5.4.2 Box and block test (BBT)

The BBT was performed to assess gross manual dexterity (42). The participants faced a rectangular wooden box that was separated into two equal compartments (29 cm wide, 17 cm high) by a partition, and 150 colored wooden blocks (2.5 cm cubes) were placed in the compartment on the paretic side. The participants were instructed to move a block, one at a time, over the partition from one compartment to the other, using the paretic hand during a 60-s trial. The test was recorded using a video camera.

BBT scores were determined by the number of accurately transferred blocks within 60 s using an offline video. Trials were not counted if the participant moved a block without their hand crossing the partition or slid a block across the partition. Trials in which multiple blocks were transferred simultaneously were counted as single points. Although the MCID for BBT has not been established, the minimal detectable change is estimated to be 5.5 blocks (43).

##### 2.5.4.3 Modified ashworth scale (MAS)

Muscle tone of the affected upper limb was graded using the MAS, which has been demonstrated as a reliable measure of spasticity (44). The measured joint motions were elbow flexion, elbow extension, forearm pronation, forearm supination, wrist palmar flexion, wrist dorsiflexion, flexion of the metacarpophalangeal (MC) joint of the index finger, and extension of the MC joint of the index finger. The assessor passively extended the target joint from a position of maximal possible flexion to maximal possible extension over a duration of approximately 1 s. The MAS score, on a 6-point scale of 0, 1, 1+, 2, 3, and 4, was determined by the resistance felt by the assessor during the passive movement (0 = “no increase in muscle tone,” 4 = “limb is rigid for flexion or extension”). Measurements were performed three times for each joint movement, and the lowest score was considered. The MCID for MAS is estimated as 0.48–0.76 with moderate to large effect sizes (45).

##### 2.5.4.4 Range of joint motion (ROM)

The passive ROM of the affected upper limb was measured using a standard goniometer according to the guidelines of the Japanese Association of Rehabilitation Medicine. The measured joint motions included shoulder flexion, shoulder abduction, shoulder external rotation, shoulder internal rotation, elbow flexion, elbow extension, forearm pronation, forearm supination, wrist palmar flexion, wrist dorsiflexion, flexion of the index-finger MC joint, and extension of the index-finger MC joint. The measurements were performed once for each movement.

### 2.6 Reporting guideline

This article was written according to the single-case reporting guidelines in Behavioral Interventions (SCRIBE) 2016 Statement (46).

## 3 Results

### 3.1 Participants

From April 2021 to February 2023, we screened the electronic care records of 130 patients admitted to Honmoku Hospital during the study period. Of these, five individuals who met the eligibility criteria were contacted for participation and provided informed consent. Three of the five individuals were unable to perform the training task due to the absence of EMG activity in any of the recorded muscles (*n* = 1), undistinguishable EMG activity patterns across movement directions (*n* = 1), or the presence of spontaneous tonic EMG activity (*n* = 1). Spontaneous tonic EMG activity reduces the accuracy of motor-state decoding. Finally, the two remaining individuals (Participants A and B) who were able to voluntarily modify their EMG activity patterns were enrolled and underwent MVR training.

Participant A, male, 52 years old, had a right thalamic hemorrhagic stroke 4 years and 1 month before enrollment. Participant B, female, 87 years old, had a left lacunar infarction of the posterior limb of the internal capsule 1 year and 6 months before enrollment. Both participants showed chronic upper-limb motor impairment. Participant A received acute treatment and rehabilitation for 6 months, and thereafter, daycare rehabilitation services twice per week for a period of 3 years and 7 months at enrollment. Participant B received acute treatment and rehabilitation for 6 months, and thereafter, daycare rehabilitation services twice per week for 1 year.

### 3.2 Implementation status

Both participants completed the planned intervention period without discontinuation. During the intervention period, neither participant received any other treatment or rehabilitation training that could have influenced the motor function outcomes. Participant A completed 40 sessions of the MVR + rehab phase. He performed a total of 1,368 repetitions of the test movements for the posture-matching test during the MVR + rehab phase (456, 384, and 528 repetitions in the wrist, hand, and mixed tasks, respectively; Supplementary Table S1). The number of repetitions of the test movements for offline decoding was reduced from three to two owing to fatigue and hypertonus in the finger flexor muscles. In addition, clinical assessments at months 4 and 7 were not performed due to absence for personal reasons. For Participant B, the number of sessions during the rehab-alone phase was reduced to seven because of the care-service usage schedule. She underwent 672 repetitions of test movements for the posture-matching test during the MVR + rehab phase for 3 months (336, 204, and 132 repetitions in the wrist, hand, and mixed tasks, respectively; Supplementary Table S1). There were no clinical AEs recorded during the intervention period.

### 3.3 Offline decoding accuracy

Figure 2 shows the temporal changes in accuracy scores obtained for each participant (Supplementary Table S2). In the single task (blue symbols) for Participant A, the mean accuracy score across the four types of movements was 86.8 at baseline (Session 1), which increased to 93.5 after completion of the MVR + rehab phase (Session 40). For Participant B, the mean accuracy score increased from 83.3 (Session 1) to 91.8 (Session 20). However, the accuracy scores exhibited substantial variability owing to factors such as electrode condition and participant fatigue, and the temporal improvements were mostly not statistically significant. Correlation analysis of temporal changes revealed a weak positive correlation only for the wrist palmar flexion score in Participant A (*r* = 0.39, *p* = 0.018). For Participant B, no significant correlations were observed for any movement; however, scores below 75 were no longer observed in later sessions. For the mixed task (red symbols), no significant changes were observed for either participant because of the short evaluation period and large variability in the measurements.

**Figure 2:**
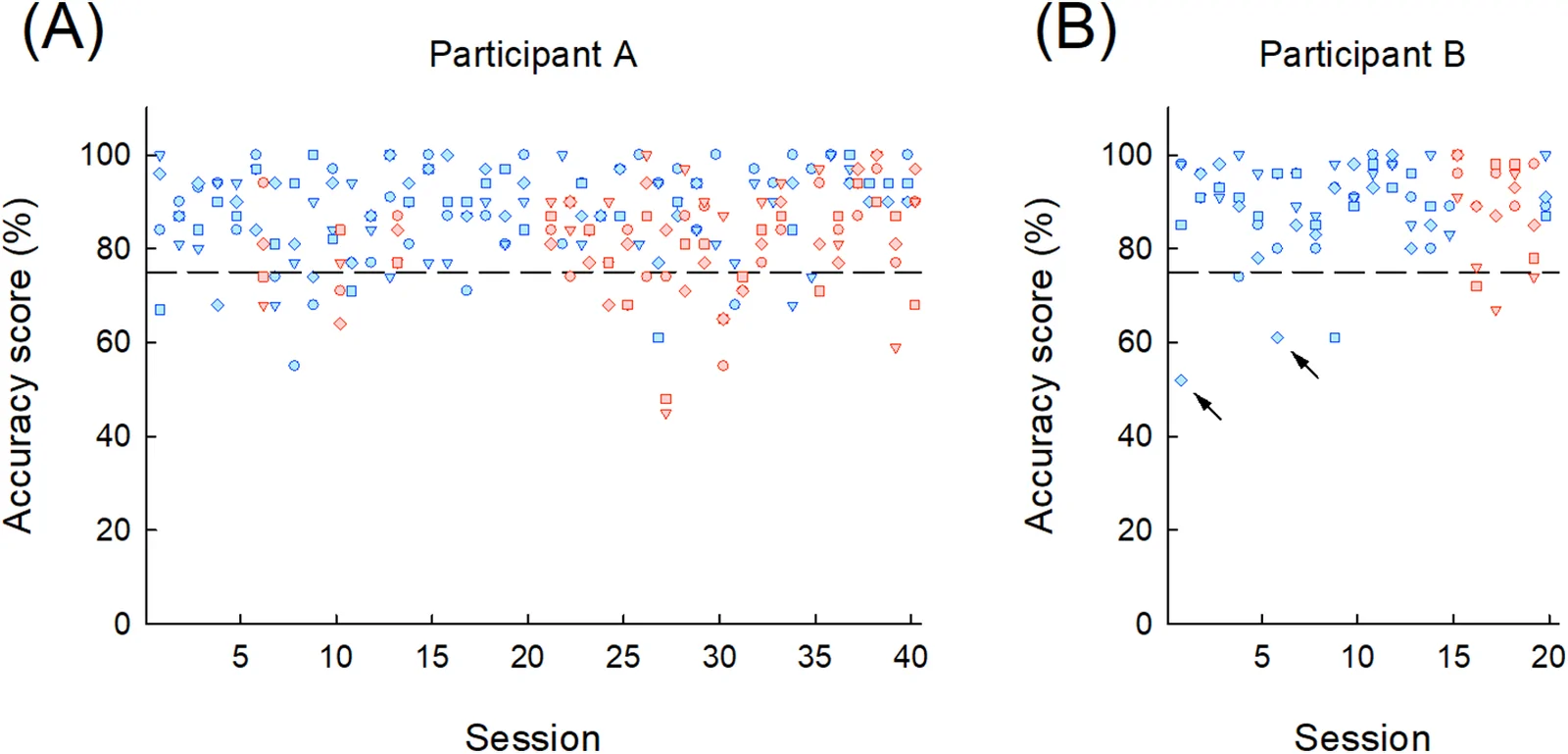
Offline decoding accuracy for MVR training in Participant A **(A)** and Participant B **(B)**. Individual accuracy scores for each test movement in the single task (blue circle: wrist palmar flexion; blue triangle down: wrist dorsiflexion; blue square: hand closed; blue diamond: hand open) and mixed task (red circle: wrist palmar flexion; red triangle down: wrist dorsiflexion; red square: hand closed; red diamond: hand open). The horizontal dashed line represents a reference accuracy score (75%).

### 3.4 Perceptions of avatar control

Participant A seemed impatient when the trial failed in the early sessions. He frequently reported a feeling of dissociation between the avatar and his own hand movements even when the avatar was successfully moved. In such cases, he visually searched for paretic hand movements. The investigator then reinstructed the participant to view the avatar on the monitor. At session 15 (month 2), he verbally reported that “I felt good today. I do not have to exert much force when the avatar moves like this.” At session 35 (month 5), he also reported that “I wasn't as tired as before.”

For Participant B, we could not obtain clear subjective reports on the training because of dysarthria. She could perform a posture-matching test while continuously observing the avatar's movements.

### 3.5 Effectiveness

#### 3.5.1 Sensorimotor function

Figure 3 shows the FMA-UE scores for each participant. For participant A (Figure 3A), the total motor score of the FMA-UE at enrollment (month 0) was 15 out of 66 points. After completion of 40 sessions of MVR + rehab (month 8), the FMA-UE motor score increased to 22 points (+7 points from month 0). In particular, the increase in scores was attributed to changes in the scores of the upper extremities (+2 points), wrist (+2 points), and hand (+3 points), whereas there was no change in the coordination/speed score (Table 1). Participant A showed severe somatosensory impairment in the affected arm according to the sensory score of the FMA-UE. The total sensation score at enrollment (month 0) was 1 point (light touch, 0 points; position, 1 point), and there was no change in the sensation score over 8 months in the MVR + rehab phase (Table 1).

**Figure 3:**
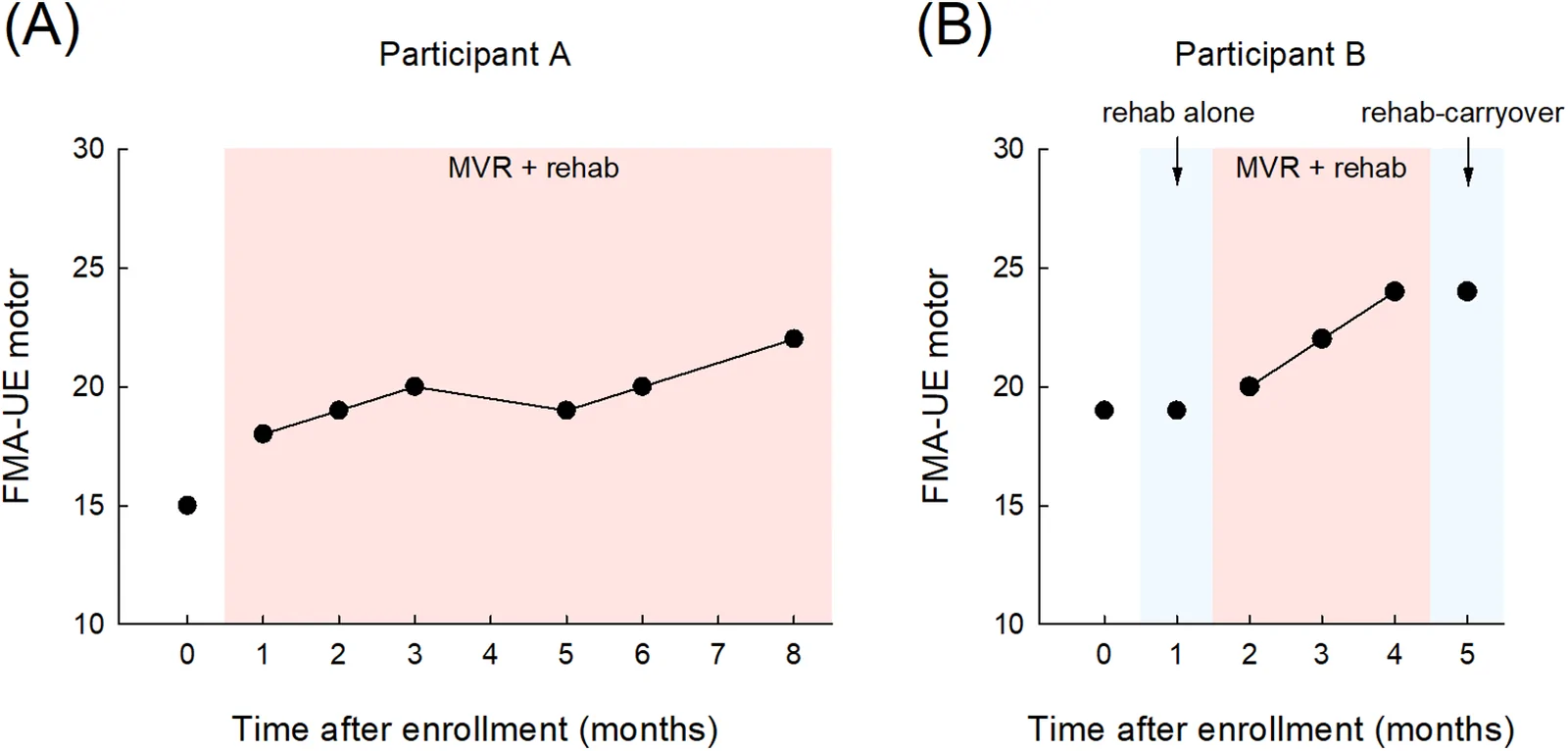
Clinical effectiveness for upper-limb motor function in Participant A **(A)** and Participant B **(B)**. Each plot indicates the total motor score of the Fugl–Meyer assessment of the upper extremity (FMA-UE).

**Table 1:** Breakdown of the motor and sensation scores of the Fugl–Meyer assessment of the upper extremity (FMA-UE).

Participant A									
	Study phase (month)								
	Enrollment	MVR + rehab							
	0	1	2	3	4	5	6	7	8
Motor									
Upper Extremity	10	10	11	10	-	10	11	-	12
Wrist	3	4	5	5	-	4	4	-	5
Hand	2	4	3	5	-	5	5	-	5
Coordination/Speed	0	0	0	0	-	0	0	-	0
Total	**15**	**18**	**19**	**20**	-	**19**	**20**	-	**22**
Sensation									
Light Touch	0	0	0	0	-	0	0	-	0
Position	1	1	1	1	-	1	1	-	1
Total	**1**	**1**	**1**	**1**	-	**1**	**1**	-	**1**

Participant B									
	Study phase (month)								
	Enrollment	rehab alone	MVR + rehab			rehab-carryover			
	0	1	2	3	4	5			
Motor									
Upper Extremity	11	11	12	12	13	13			
Wrist	3	3	3	3	3	3			
Hand	5	5	5	7	8	8			
Coordination/Speed	0	0	0	0	0	0			
Total	**19**	**19**	**20**	**22**	**24**	**24**			
Sensation									
Light Touch	4	4	4	4	4	4			
Position	8	8	8	8	8	8			
Total	**12**	**12**	**12**	**12**	**12**	**12**			

Total motor score in Table 1 provides source data for Figure 3. Bold values indicate total scores for the motor and sensory domains. -, not assessed.

For Participant B (Figure 3B), the FMA-UE motor score at enrollment (month 0) was 19 out of 66. After completion of the rehab alone phase (month 1), there was no change in the motor score. Thereafter, the motor score increased over the MVR + rehab phase (month 1 vs. months 2–4), reaching 24 points at completion of the MVR + rehab phase (month 4) (+5 points from month 1). In particular, the increase in scores depended on changes in the scores of the upper extremities (+2 points) and hand (+3 points) (Table 1). The motor score remained constant over the rehab-carryover phase in which MVR training was withdrawn (months 4 vs. 5), and no additional changes in the scores were observed. The total sensation score at enrollment was 12 points (perfect score) and remained constant over all phases, indicating normal upper extremity somatosensory function (Table 1).

#### 3.5.2 Gross manual dexterity

Figure 4 shows the BBT scores for each participant. For Participant A, the BBT score at month 0 was 2 blocks (Figure 4A). At month 8, he was able to perform the task better, with greater hand opening while grasping and releasing the block (Supplementary Video S2). His BBT score increased from two blocks in month 0 to five blocks in month 8. The BBT score of participant B increased from 4 blocks in month 0–5 blocks in month 1 and further increased to 12 blocks in month 4 (+7 blocks from month 1) (Figure 4B). At month 4, she was able to grasp the block with the fingertips, and the speed of transfer increased compared with that at month 0 (Supplementary Video S3). Her BBT score decreased by two blocks in month 5.

**Figure 4:**
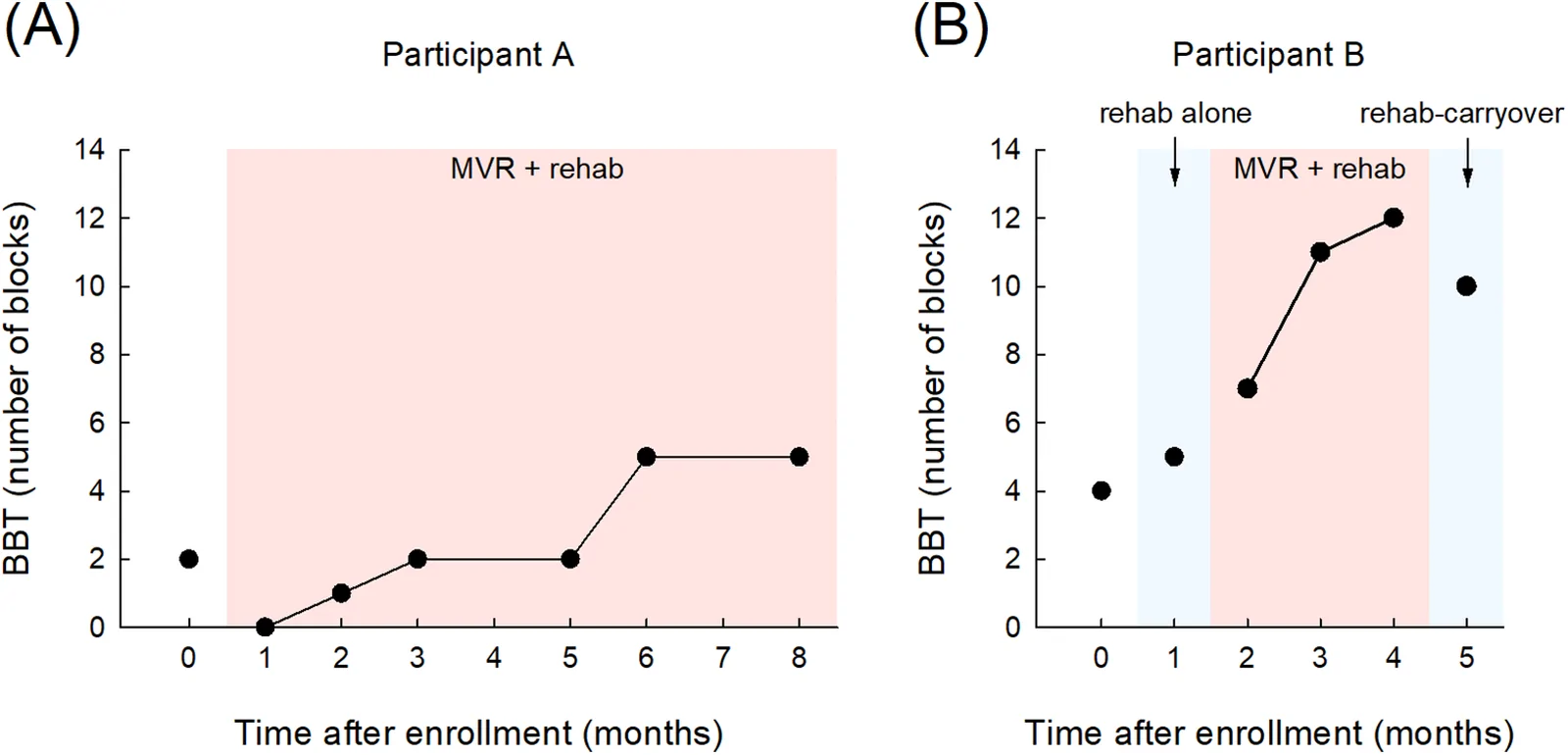
Clinical effectiveness for gross manual dexterity in Participant A **(A)** and Participant B **(B)**. Each plot indicates scores of the box and block test.

#### 3.5.3 Muscle tone

The MAS scores are listed in Table 2. For Participant A, the MAS scores for both flexor and extensor muscles involving the wrist and finger joints improved by 1 point in month 8 compared to month 0 (Table 2). In Participant B, the MAS scores for the wrist and finger flexors improved by 2 grades during the MVR + rehab phase, whereas there was no change in the MAS scores during the rehab alone phase (Table 2). During the rehab-carryover phase, such improvement in the MAS score was maintained.

**Table 2:** Modified Ashworth scale (MAS).

Participant A									
Joint motion	Study phase (month)								
	Enrollment	MVR + rehab							
	0	1	2	3	4	5	6	7	8
Elbow									
Flexion	1	1	1	1+	-	1	1	-	0
Extension	1+	1+	2	1+	-	1+	2	-	2
Forearm									
Pronation	0	0	0	0	-	0	0	-	0
Supination	2	2	2	1+	-	1+	1+	-	1+
Wrist									
Palmar flexion	1+	1+	1	1	-	1	1	-	1
Dorsiflexion	2	2	1+	1+	-	1+	1+	-	1+
Finger									
Flexion	1+	1+	1+	1	-	1	1	-	1
Extension	2	2	2	1+	-	1+	1+	-	1+

Participant B									
Joint motion	Study phase (month)								
	Enrollment	rehab alone	MVR + rehab			rehab-carryover			
	0	1	2	3	4	5			
Elbow									
Flexion	2	2	2	2	2	2			
Extension	1+	1+	1+	1	1	1			
Forearm									
Pronation	0	0	0	0	0	0			
Supination	2	2	2	2	1	1			
Wrist									
Palmar flexion	1	1	1	1	1	1			
Dorsiflexion	2	2	1	1	1	1			
Finger									
Flexion	1	1	1	1	1	1			
Extension	2	2	2	1	1	1			

For the finger joint, motions of the metacarpophalangeal (MC) joint of the index finger were assessed. -, not assessed.

#### 3.5.4 ROM

The ROM values are summarized in Table 3. In Participant A, the ROM increased by 15° for wrist dorsiflexion and by 10° for finger flexion and extension from month 0 to month 8. In Participant B, the ROM did not change significantly throughout the intervention period, except for the ROM for wrist palmar flexion, which was reduced by 15° in months 3–5 from month 0.

**Table 3:** Range of joint motion (ROM) in degree at upper-limb joints.

Participant A									
Joint motion	Study phase (month)								
	Enrollment	MVR + rehab							
	0	1	2	3	4	5	6	7	8
Shoulder (°)									
Flexion	70	80	80	90	-	90	90	-	90
Abduction	75	75	75	80	-	80	90	-	90
External rotation	15	15	15	15	-	15	15	-	15
Internal rotation	50	50	50	50	-	50	50	-	50
Elbow (°)									
Flexion	115	115	115	115	-	115	115	-	115
Extension	0	0	0	0	-	0	0	-	0
Forearm (°)									
Pronation	90	90	90	90	-	90	90	-	90
Supination	45	45	45	50	-	50	55	-	55
Wrist (°)									
Palmar flexion	55	55	55	60	-	60	60	-	60
Dorsiflexion	35	50	50	50	-	50	50	-	50
Finger (°)									
Flexion	60	70	70	70	-	70	70	-	70
Extension	20	30	30	30	-	30	30	-	30

Participant B									
Joint motion	Study phase (month)								
	Enrollment	rehab alone	MVR + rehab			rehab-carryover			
	0	1	2	3	4	5			
Shoulder (°)									
Flexion	80	80	80	80	80	80			
Abduction	70	80	80	80	80	80			
External rotation	15	15	15	15	15	15			
Internal rotation	50	50	50	50	50	50			
Elbow (°)									
Flexion	100	100	100	100	100	100			
Extension	−20	−20	−20	−20	−20	−20			
Forearm (°)									
Pronation	90	90	90	90	90	90			
Supination	30	30	30	30	30	30			
Wrist (°)									
Palmar flexion	85	85	80	70	70	70			
Dorsiflexion	45	45	45	50	50	50			
Finger (°)									
Flexion	90	90	90	90	90	90			
Extension	20	20	20	20	20	20			

For the finger joint, motions of the metacarpophalangeal (MC) joint of the index finger were assessed. -, not assessed.

## 4 Discussion

Our findings indicate that long-term MVR training can be feasible in clinical settings for individuals with chronic motor paralysis after stroke. Moreover, long-term MVR training with conventional rehabilitation therapies may be safe and associated with improvements in upper-limb motor function in such individuals.

### 4.1 Safety

No adverse clinical events were reported by the participants in this study. A recent systematic review on the use of VR for motor rehabilitation after stroke summarized the occurrence of clinical AEs, including upper-limb fatigue, dizziness, headache, general fatigue, falls, pain, eye strain, blurred vision, and shoulder soreness, regardless of whether they were related to VR training; however, the pooled AEs have not been systematically analyzed due to different comparisons, different intervention approaches, and events reported (19). A recent study using the MVR intervention approach in a small participant population reported skin irritation from the electrodes (30). However, we consider that the MVR interventions to be generally safe because no major AEs have been reported by most studies included in the review (46 out of 59 studies) (19). The EMG electrode-related skin issues were improved by careful precautions, such as more rigorous cleaning procedures and skin aftercare using cream (30), and the occurrence of cybersickness experienced in a non-immersive VR environment using a laptop screen, as in the present study, is low compared to fully immersive VR using a head-mounted display (47).

### 4.2 Clinical feasibility

Regarding clinical feasibility, the results suggest that MVR training can be implemented for post-stroke motor rehabilitation in outpatient clinics. Furthermore, the MVR system may be available to a wider range of user populations who have experienced a stroke than previously expected.

First, non-invasive EMG recording using a wearable wireless device enables users to engage in a task with less physical restraint and without occupying space in the clinical environment. In addition, using such a system, the training setup, including electrode placement and device setup, could be completed within approximately 10 min. Given its advantages of operability and ease of preparation, it is feasible to implement this system as an adjunct to standard rehabilitation in clinical settings.

Second, the ability to modify EMG activity may provide useful information for screening target populations for MVR training. The pre-screening results suggest that MVR training can be applied to individuals who are capable of modulating their EMG activity, even if the accuracy score for one of the antagonistic movements in a single-joint task is as low as approximately 50% (Supplementary Table S2). The accuracy score was decreased by the post-stroke abnormality of spatiotemporal muscle coactivation patterns (48–50) or the reduction of EMG activity due to motor paralysis (Supplementary Figure S1). Practically, pre-evaluated accuracy scores in possible candidates who were not assigned to the MVR training due to the inability to perform posture-matching tests were lower than those reported in able-bodied individuals (∼95%) (27, 33) and individuals with upper-limb amputation (26, 34). In contrast, the aforementioned criterion for the accuracy score is more lenient than that used in a previous study (i.e., 75% and higher accuracy) (30). The training task in the present study was simple and might not require high decoding accuracy to perform the training task, compared to the gamified tasks in the previous study. These lenient criteria may contribute to expanding the eligible population for MVR training.

Third, MVR training may apply to individuals with somatosensory deficits after stroke, based on the implementation status of Participant A, who had severe somatosensory impairments involving both tactile sensation and proprioception. It should be noted that he also experienced a feeling of dissociation between the avatar's and his own hand movements in the early sessions, consistent with experimentally induced proprioceptive-visual mismatch perception in healthy individuals (51). Although the mechanistic elaboration of his experience is speculative due to a lack of evidence, the somatosensory deficits and sensorimotor mismatch perception might have impeded task performance and the progress of training to a greater or lesser extent based on his lower task progress (Supplementary Table S1) and functional gain (discussed later) compared to Participant B, whose somatosensory function was normal. Additionally, Participant A was aware of the increased controllability of the virtual hand and experienced positive feelings during the middle sessions. Since such an experience did not accompany improvement in somatosensory function, we speculate that changes in the visuo–proprioceptive–motor integration process occurred through proprioceptive recalibration and/or increased somatosensory feedback of improved upper-limb activity (52). Thus, we interpret that somatosensory impairments after stroke need to be considered but would not be problematic as long as individuals can adapt to the altered feedback gain during a task.

Fourth, both participants completed the planned training sessions under the personal adjustment of the repetition numbers and challenge level required for the task. Feedback from Participant A during the MVR training suggested that he required high effort to perform the task in the early training sessions, but the subjective effort diminished over the sessions. Participant A completed an increased number of movement repetitions and challenging tasks in the latter half of the training period. Thus, adjusting the number of test movement repetitions for offline decoding, as in Participant A, and directly evaluating the feasibility of the posture-matching test were also considered important for expanding the eligibility criteria for VR-based training. In contrast, participant B was able to perform an increased number of movement repetitions in the wrist/hand task in the first month of the MVR + rehab phase, maintain the repetition number thereafter, and smoothly transfer the task level from the wrist/hand task to the mixed task during the last 1 month of the MVR + rehab phase.

### 4.3 Effectiveness

Although clinical effectiveness was not determined, long-term MVR training plus conventional rehabilitation care may have provided one of the two participants with clinically significant benefits for improvement of motor function, that is, an increase in the FMA-UE motor score exceeding the estimated MCID. Longitudinal large-scale cohort surveys have suggested that functional outcomes and gains in post-stroke recovery largely depend on time, clinical severity of paralysis, and age (6, 53–55). At enrollment, the levels of upper-limb motor impairments in both participants were considered severe (56), the clinical signs were stable over a long period after stroke onset; and Participant B was over 85 years old. Therefore, spontaneous recovery in these participants would reach a plateau, and training responsiveness would be poor. However, the results showed that motor functional changes exceeding the MCID threshold occurred during MVR + rehab even in a participant who was approximately 4 years post-stroke. These results imply that MVR training may target individuals with a wide range of time progression, severity of paralysis, and age, when appropriate.

The present study configured MVR training with a short duration (30 min per session) and a small number of movement repetitions (24–48 repetitions per session). Individuals with chronic moderate or severe paralysis after stroke require long-term intensive therapy to achieve clinically significant benefits (57). Although ideal, increasing the duration of intensive rehabilitation services in individuals at the chronic stage is not feasible under the medical and long-term care insurance systems in Japan. Therefore, MVR training could be incorporated into the clinical setting under limited situations and continued for at least 3 months.

Since the functional gain of the FMA-UE motor score was larger over the MVR + rehab phase than the rehab alone phase in Participant B, although the changes in the FMA-UE motor score over the MVR + rehab phase in this participant did not reach the MCID threshold. Thus, long-term MVR training plus conventional rehabilitation care might have clinical potential for the improvement of motor function, compared to conventional rehabilitation care alone. The different functional gain may be explained by the addition of MVR training or simply by the increased duration of hand motor activity. For instance, the duration of each session in the MVR + rehab phase was increased compared to the rehab alone phase. In addition, active hand exercises were administered during the MVR + rehab phase but not during the rehab alone and rehab-carryover phases. Therefore, MVR training provides increased opportunities for individuals to engage in training within a restricted training time, which is crucial for motor rehabilitation after chronic stroke (58).

Although somatosensory function did not change over time in either participant, residual somatosensory function after stroke could affect motor performance (59, 60) and the functional gain of post-training hand motor outcomes (61). As previously mentioned, Participant A had severe somatosensory impairment. Such altered sensorimotor integration may have caused the slower task progress in repetition numbers per session and task level over sessions in Participant A than in Participant B, whose somatosensory function was normal.

Post-training changes in motor function were accompanied by those in gross manual dexterity, muscle tone, and ROM. However, it remains unclear whether these changes are related to or occur independently of each other. For both participants who showed mild-to-moderate muscle hypertonicity, especially for the flexor muscles, a clinically significant reduction in the MAS score was observed in the early sessions of the MVR + rehab phase. It remains controversial whether improved muscle hypertonicity directly contributes to improved motor function because of the complicated mechanisms underlying post-stroke spasticity (62–65). Especially at the chronic stage, in addition to neural factors (e.g., motoneuronal hyperexcitability), non-neural factors (e.g., changes in the tissue properties of muscle tendons spanning the joint) would substantially contribute to exaggerated stretch reflexes and muscle hypertonus (66). Therefore, if prolonged muscle weakness and hypertonicity would secondarily induce ROM limitation, removal of the deconditioning states by VR + rehabilitation may expand ROM and improve movement control, indirectly contributing to the improvement of gross manual dexterity for performing a hand motor task (67, 68). However, at least for Participant B, who showed improved motor performance without changes in ROM, we presume that other factors (e.g., neural factors) contributed to post-training motor improvement.

### 4.4 Limitations

This study had some limitations. First, the present study does not provide solid evidence of clinical efficacy of long-term MVR training because it was designed to assess feasibility and clinical potential. The small sample size, lack of randomization, absence of blinded assessment, selection bias, limited baseline observations, and restricted generalizability limit the strength and interpretation of the findings.

Second, whether the observed changes are related to MVR training itself or other factors. As aforementioned, conventional rehabilitation care was provided throughout all phases, making it difficult to isolate the specific contributions of MVR training. Therefore, the present study cannot clarify possible confounding effects of previous or concurrent interventions, deconditioning effects before participation, an increased dose or repetitions of upper-limb active motor activity, increased treatment time, therapist attention, other nonspecific factors, or their combination.

Third, the participants’ clinical characteristics were heterogeneous since this was an exploratory study. Thus, what participant characteristics affect feasibility, safety, and responsivity to MVR training remains unclear.

Fourth, the limited baseline data points and the lack of stable baseline trends restrict the ability to interpret phase-dependent changes and limit causal inferences regarding intervention effects. Since follow-up assessment was not performed after the MVR + rehab phase, whether the intervention effect was maintained remains unclear. No predefined criteria for progression of task difficulty were established, and the task difficulty was not systematically increased during the intervention period. Determining optimal training conditions (e.g., task progression, dose, intensity, and frequency) of MVR training remains an unresolved issue.

Therefore, clinical efficacy of MVR training and the related factors influencing its responsivity need to be established in adequately powered controlled trials.

## 5 Conclusion

This exploratory single-case study demonstrated feasibility and preliminary clinical potential of the long-term use of the MVR system for the two participants with chronic motor paralysis after stroke. However, adequately powered controlled trials are required before conclusions regarding its effectiveness can be drawn. Our results also suggest that MVR training may potentially target a wide population of stroke survivors, regardless of the long period after onset, severe sensorimotor impairments, and advanced age.

## Data Availability

The original contributions presented in the study are included in the article/Supplementary Material, further inquiries can be directed to the corresponding author.

## Supplementary material

Supplementary Figure 1 Representative example of electromyographic activity during the hand opening (**A–C**) and closing (**D–F**) movements of the recording sessions in the sessions 1 (**A, D**), 6 (**B, E**) and 20 (**C, F**) obtained from participant B. The electromyographic activity was recordedfrom 8 muscles of the affected upper limb: biceps brachii (BB), triceps brachii (TB), brachioradialis (BR), flexor carpi radialis (FCR), flexor digitorum superficialis (FDS), extensor carpi radialis (ECR), extensor carpi ulnaris (ECU), and extensor digitorumcommunis (EDC). A shaded area indicates a movement period.

Supplementary Table S1 Number of repetitions for the posture-matching test. -, not performed.

Supplementary Table S2 Source data for Figure 2. -, not performed.

Supplementary Table S3 Source data for Figure 4. -, not performed.

Supplementary Video S1 Video of Participant B while performing the single hand task (hand closing) of the posture-matching test. The video was recorded in session 1.

Supplementary Video S2 Video of Participant A during the box and block test. The video was recorded at enrollment (month 0) and at month 8.

Supplementary Video S3 Video of Participant B during the box and block test. The video was recorded at enrollment (month 0) and at month 4.
